# Tuberculous Myopericarditis With Constrictive Physiology: A Case Report Using a Multimodality Approach

**DOI:** 10.7759/cureus.103737

**Published:** 2026-02-16

**Authors:** Oswaldo Alejandro Angel Bran, Marco Antonio Ponce-Gallegos, Diego Artemio Valadez Villegas, Gustavo Salinas Arteaga, José Luis Briseño de la Cruz

**Affiliations:** 1 Cardiology, Instituto Nacional de Cardiología Ignacio Chávez, Mexico City, MEX

**Keywords:** constrictive myopericarditis, constrictive tuberculous pericarditis, extrapulmonary tuberculosis (eptb), multimodality cardiac imaging, right and left heart catheterization

## Abstract

Tuberculous pericarditis is a rare but important cause of constrictive pericarditis in developed countries, while remaining a significant public health concern in endemic regions. Myocardial involvement is uncommon and often underrecognized. A previously healthy 47-year-old man presented with a five-year history of progressive bilateral lower-limb edema and exertional dyspnea, with marked deterioration over the last five months, including severe fatigue and involuntary weight loss of 8 kg. On admission, imaging revealed biventricular systolic dysfunction, pericardial thickening with calcifications, and bilateral pleural effusions. Pleural fluid analysis demonstrated an exudative effusion with markedly elevated adenosine deaminase (ADA) levels (130 U/L), consistent with tuberculous pleuritis. Cardiac magnetic resonance imaging confirmed myopericarditis with diffuse pericardial thickening (up to 6 mm) and late gadolinium enhancement. Right heart catheterization demonstrated findings consistent with constrictive physiology. Given the presence of severe ventricular dysfunction, atrial fibrillation, and active extrapulmonary tuberculosis, pericardiectomy was deferred. Antituberculous therapy and guideline-directed medical treatment for heart failure were initiated. At three-month follow-up, the patient demonstrated mild functional improvement and continued on a standard six-month antituberculous regimen, which was ongoing at the time of reporting. In endemic areas, tuberculosis should be considered an important cause of constrictive pericarditis in patients with unexplained pericardial disease. Multimodality imaging is essential for accurate diagnosis and for guiding management, especially in complex presentations involving both the pericardium and myocardium. This case illustrates the diagnostic value of a multimodality approach and the management strategy in a high-risk surgical candidate.

## Introduction

Tuberculous pericarditis, although uncommon in developed countries, remains a significant cause of morbidity and mortality worldwide, particularly in regions with high tuberculosis (TB) prevalence. Tuberculous pericarditis is found in approximately 1% of all autopsied TB cases but represents the most common cause of pericarditis in regions where TB remains a major public health problem [1]. By contrast, in developed countries, TB is now a rare cause of constrictive pericarditis, reported in less than 1% to 5.6% of cases. The predominant etiologies in these regions are now idiopathic, prior cardiac surgery, and radiation therapy, with one major center reporting 80% of cases falling into these categories [2]. Constrictive pericarditis is a serious complication of tuberculous pericarditis, often presenting with an insidious onset of heart failure symptoms, which may be challenging to diagnose.

Pericardial involvement in TB is thought to occur through lymphatic spread from mediastinal lymph nodes, direct extension from adjacent pleuropulmonary disease, or hematogenous dissemination. Constrictive pericarditis is a serious complication of tuberculous pericarditis, often presenting with an insidious onset of heart failure symptoms, which may be challenging to diagnose. Diagnosis is further complicated by the paucibacillary nature of extrapulmonary TB, frequently limiting microbiological confirmation and necessitating an integrated diagnostic approach.

In this case report, we present a 47-year-old patient with a prolonged history of lower-extremity edema and progressive dyspnea who was ultimately diagnosed with constrictive pericarditis and myocarditis secondary to *Mycobacterium tuberculosis*. This case highlights the importance of considering TB as a potential etiology in patients with unexplained pericardial or myocardial disease, particularly in endemic areas, and underscores the utility of multimodality imaging and invasive hemodynamic assessment in establishing a definitive diagnosis.

## Case presentation

A previously healthy 47-year-old Mexican man, born and residing in a rural area of Mexico, presented with a five-year history of progressive bilateral indurated lower-extremity edema and exertional dyspnea. He lived in a crowded household with five family members in a small dwelling and was currently unemployed, having previously worked in manual labor occupations, including construction and carpentry. He denied prior incarceration, military service, or known direct contact with individuals diagnosed with TB.

He was initially diagnosed with heart failure and treated with diuretics and dietary salt restriction, with intermittent symptomatic improvement. However, over the preceding five months, his clinical condition deteriorated significantly, with the development of extreme fatigue, dyspnea on minimal exertion, involuntary weight loss of 8 kg, and persistent night sweats. He denied fever, hemoptysis, or chronic cough.

**Figure 1:**
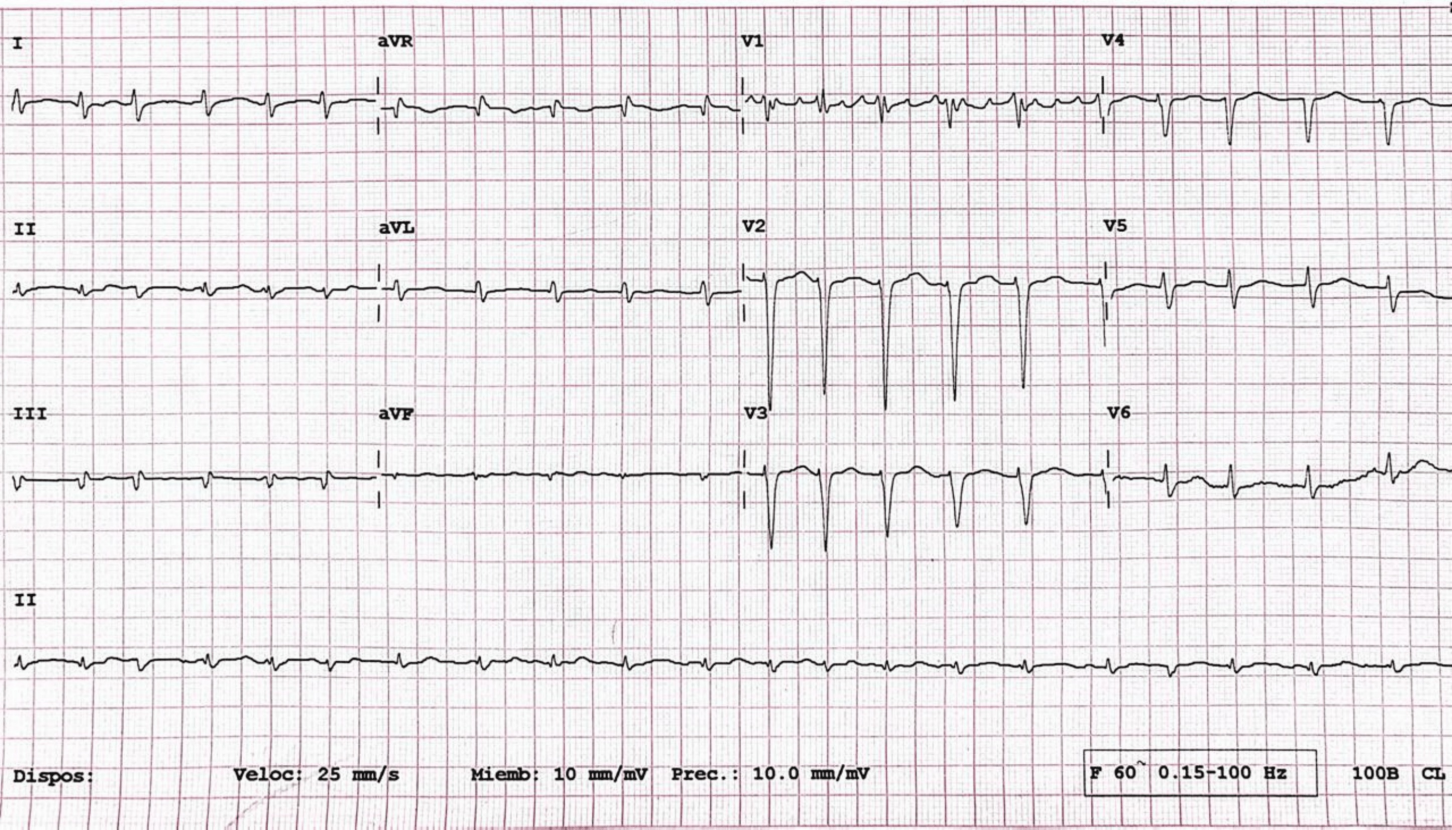
12-lead electrocardiogram demonstrating atrial fibrillation with rapid ventricular response

Upon initial assessment, the patient presented with tachycardia (110 bpm), tachypnea (21 breaths/min), hypotension (95/61 mmHg), and oxygen desaturation (90%). The physical examination revealed elevated jugular venous pressure, bilateral pleural effusions, irregular heart sounds with a mitral regurgitant murmur, ascites, and hyperpigmented lower-limb edema. The electrocardiogram (Figure 1) demonstrated atrial fibrillation with a rapid ventricular response. Initial laboratory investigations (Table 1) showed markedly elevated NT-proBNP (9,101 pg/mL) and high-sensitivity troponin T (134 pg/mL).

**Table 1:** Laboratory results at the time of admission WBC: white blood cells, RBC: red blood cells, Hgb: hemoglobin, Hct: hematocrit, MCV: mean corpuscular volume, MCH: mean corpuscular hemoglobin, MCHC: mean corpuscular hemoglobin concentration, BUN: blood urea nitrogen, AST: aspartate aminotransferase, ALT: alanine aminotransferase, HBsAg: hepatitis B surface antigen, HIV Ag/Ab: HIV antigen/antibody, NT-Pro BNP: N-terminal pro–B-type natriuretic peptide, HBsAg: hepatitis B surface antigen.

Laboratory parameters	Value	Normal value
Complete blood count		
WBC	6.78 K/µL	3.84–9.79 K/µL
RBC	4.18 million cells/µL	4.3–6.1 million cells/µL
Hgb	10.5 g/dL	12–16 g/dL
Hct	34%	35.4–49.4 %
MCV	81.3 fL	84.4–100 fL
MCH	25.1 pg	27.1–33.5 pg
MCHC	30.9 g/dL	31.6–34.8 g/dL
Platelets	326 K/µL	147–384 K/µL
Neutrophils	5.28 K/µL	1.71–6.48 K/µL
Lymphocytes	0.6 K/µL	0.99–3.25 K/µL
Monocytes	0.73 K/µL	0.19–0.71 K/µL
Eosinophils	0.12 K/µL	0.02–0.32 K/µL
Basophils	0.05 K/µL	0–0–09 K/µL
Renal function test		
BUN	43.5 mg/dL	6–20 mg/dL
Creatinine	1.81 mg/dL	0.7–1.2 mg/dL
Sodium	135 mmol/L	136–145 mmol/L
Potassium	3.3 mmol/L	3.5–5.1 mmol/L
Chloride	98 mmol/L	98–107 mmol/L
Liver function test		
Albumin	2.42 g/dL	3.9–4.9 g/dL
AST	25 U/L	10–50 U/L
ALT	12.4 U/L	10–50 U/L
Alkaline phosphatase	134 U/L	40–129 U/L
Total bilirubin	0.68 mg/dL	0–1.4 mg/dL
Cardiac biomarkers		
NT-Pro BNP	9101 pg/mL	5–173 pg/mL
High-sensitivity troponin T	134 pg/mL	3–14 pg/mL
Other laboratory parameters		
Random glucose	97 mg/dL	74–106 mg/dL
HIV Ag/Ab	Negative	-
HBsAg	Negative	-

**Figure 2:**
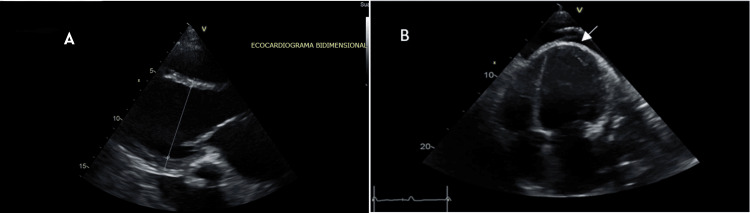
Two-dimensional transthoracic echocardiography (A) Parasternal long-axis echocardiographic view demonstrating a dilated left ventricle. (B) Apical four-chamber echocardiographic view demonstrating biatrial enlargement, thickened pericardium, and a small pericardial effusion (8 mm), without signs of tamponade physiology.

The patient was admitted for further evaluation. A chest X-ray demonstrated pericardial calcifications, which were subsequently characterized in greater detail by chest computed tomography (CT). Transthoracic echocardiography (Figure 2) performed in the setting of atrial fibrillation demonstrated left ventricular (LV) and biatrial dilatation, global LV hypokinesia with a left ventricular ejection fraction (LVEF) of 28%, calculated using the biplane Simpson method, and right ventricular (RV) systolic dysfunction. Doppler assessment of mitral inflow showed an E-wave velocity of 0.88 m/s. Tissue Doppler imaging revealed preserved septal and lateral e′ velocities (16 cm/s each), along with the presence of a septal bounce, findings supportive of constrictive physiology despite the arrhythmic rhythm. Moderate-to-severe mitral regurgitation was identified, with structurally normal mitral valve leaflets, preserved opening, and restricted closure. Quantitative parameters included a vena contracta of 0.6 cm, regurgitant volume of 25 mL, and regurgitant fraction of 46%. Additionally, a thickened and calcified pericardium was observed, associated with a mild pericardial effusion measuring up to 8 mm without hemodynamic compromise, as well as bilateral pleural effusions.

**Figure 3:**
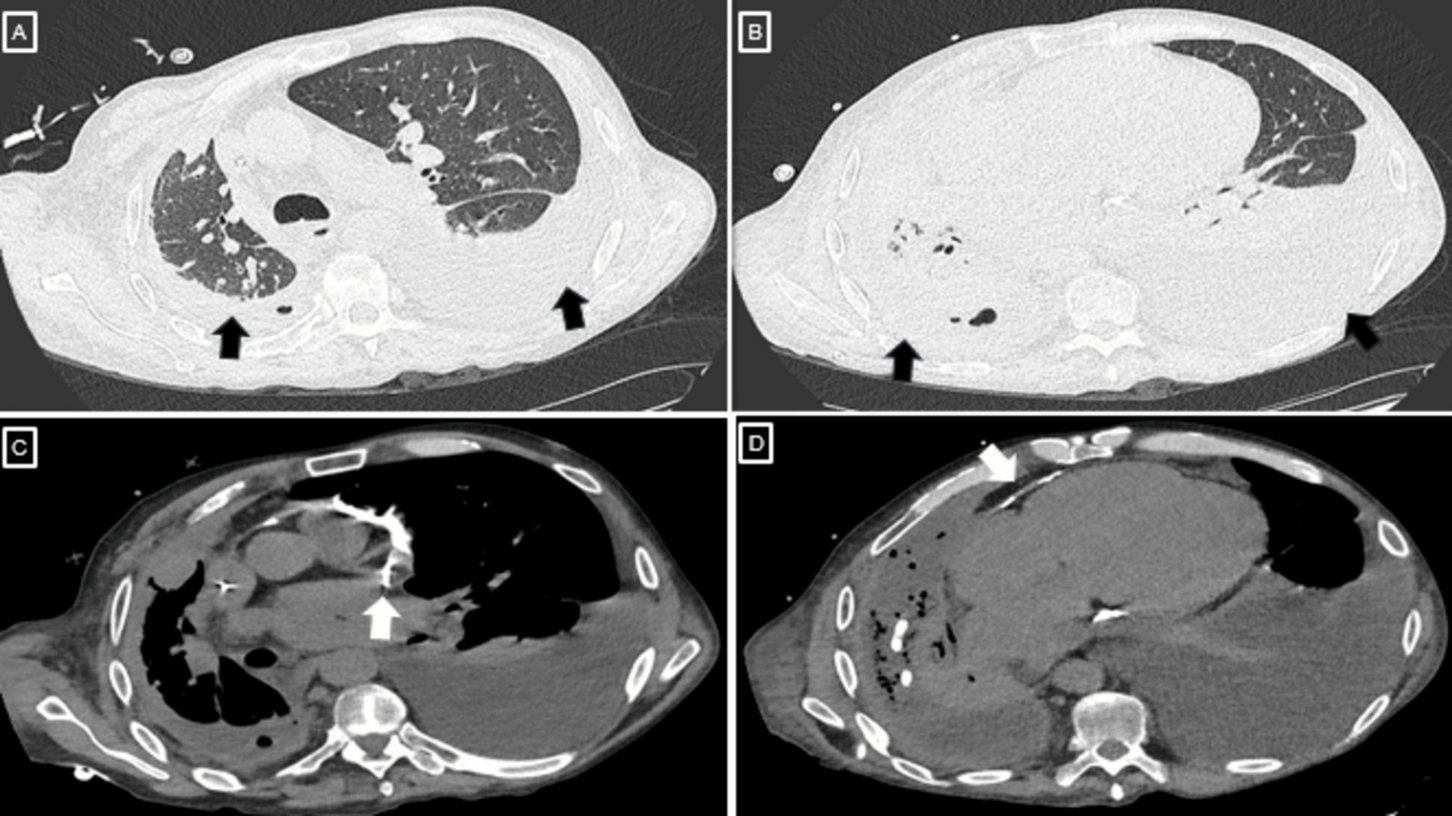
Chest computed tomography (CT) images (A, B) Axial CT views showing bilateral pleural effusions (black arrows). (C, D) Axial CT views demonstrating diffuse pericardial calcifications (white arrows) and cardiomegaly.

Subsequent chest CT (Figure 3) revealed global cardiomegaly and a low-attenuation pericardial effusion (5 Hounsfield units), with separation of the pericardial layers up to 17 mm along the RV free wall, associated with diffuse pericardial calcification. The lung parenchyma showed a mosaic attenuation pattern with interlobular septal thickening, predominantly affecting the right lung and associated with reduced right hemithorax expansion. The left hemithorax demonstrated a pleural effusion causing near-complete atelectasis of the left lower lobe, sparing the anteromedial and superior segments. No pulmonary consolidation was identified. Incidental fractures of the T6 and T11-T12 vertebral bodies were also noted.

Pleural fluid analysis (Table 2) demonstrated exudative characteristics according to Light’s criteria, with elevated lactate dehydrogenase (LDH) levels and very low pleural glucose, consistent with advanced inflammatory pleural disease. Microbiological cultures were negative. Given the epidemiological context, pleural fluid profile, and clinical presentation, adenosine deaminase (ADA) was measured and found to be markedly elevated at 130 U/L. GeneXpert MTB/RIF testing for *M. tuberculosis* was negative, and cytological analysis excluded malignancy.

**Table 2:** Comprehensive pleural fluid analysis Interpretation is based on Light’s criteria. ADA, adenosine deaminase; LDH, lactate dehydrogenase; AFB, acid-fast bacilli; MTB, *Mycobacterium tuberculosis*; RIF, rifampicin.

Parameter	Value	Interpretation
Aspect	Serous	Consistent with exudate
Pleural Glucose	10 mg/dL	Low
Pleural LDH	764 U/L	Elevated
Pleural Protein	1.58 g/dL	Low
Pleural Albumin	0.07 g/dL	Low
Pleural Cholesterol	7.34 mg/dL	-
Serum Glucose	79 mg/dL	Normal
Serum LDH	221 U/L	Normal
Serum Protein	4.44 g/dL	Mildly reduced
Pleural/Serum Protein Ratio	0.35	Below Light’s criterion threshold
Pleural/Serum LDH Ratio	3.45	Exudative by Light’s criteria
Leukocytes	5300 cells/mm³	-
Pleural Ziehl–Neelsen	Negative	No AFB detected
Pleural Bacterial Culture	No growth	Negative
Pleural Lowenstein-Jensen Culture	No growth at 6 weeks	Negative
Pleural Thioglycolate Culture	No growth	Negative
ADA	130 U/L	Markedly elevated
Pleural GeneXpert MTB/RIF	Negative	TB not excluded
Cytology	Negative	Malignancy excluded
Light’s Criteria	-	Exudative pleural effusion

**Figure 4:**
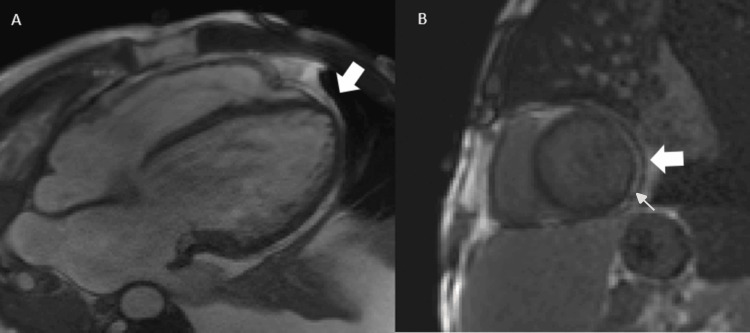
Cardiac magnetic resonance imaging (CMR) (A) Four-chamber cine sequences demonstrated a global increase in pericardial thickness (white arrow), measuring up to 6 mm in the region surrounding the left ventricle. (B) Short-axis late gadolinium enhancement (LGE) sequence showing pericardial enhancement (large white arrow) and lateral subepicardial left ventricular (LV) enhancement consistent with myocardial involvement (small white arrow). These findings are consistent with myopericarditis.

**Figure 5:**
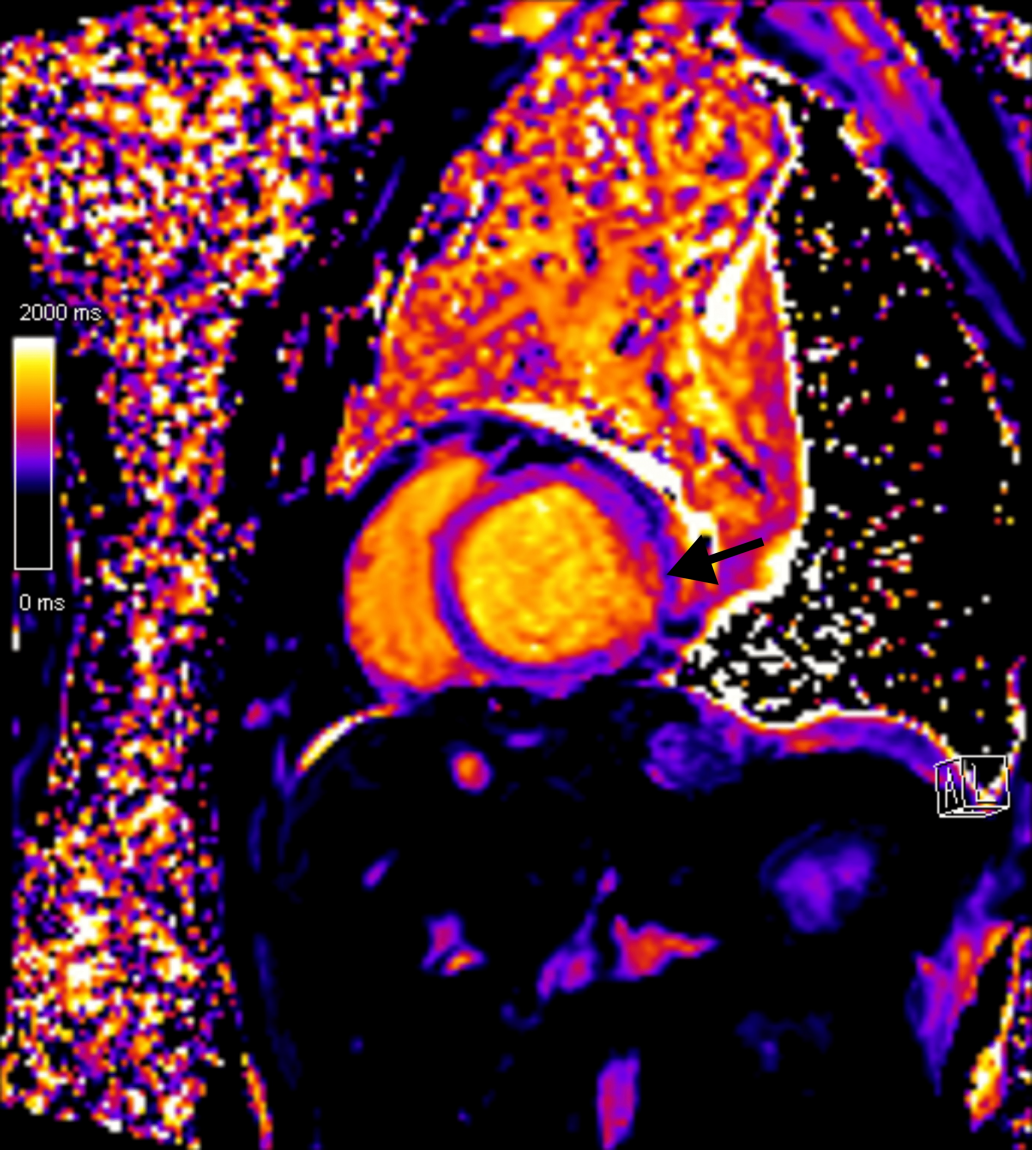
Native T1 mapping cardiac MRI sequence Increased native T1 mapping values in the inferolateral segment. MRI, magnetic resonance imaging.

In the setting of significant ventricular dysfunction, pericardial effusion with pericardial thickening, and high clinical suspicion of extrapulmonary pleural TB with probable pericardial involvement, cardiac magnetic resonance (CMR) imaging was performed (Figure 4). CMR confirmed myopericarditis, demonstrating marked pericardial thickening up to 6 mm surrounding the left ventricle and septated bilateral pleural effusions. Native T1 mapping (Figure 5) showed increased values in the inferolateral segment (1157 ms). T2-weighted and post-contrast sequences revealed subepicardial late gadolinium enhancement (LGE) in the lateral wall of the left ventricle. Biventricular systolic dysfunction was present, with an LVEF of 32%, LV mass of 124 g, LV end-diastolic volume of 224 mL, stroke volume of 72 mL, and RV ejection fraction of 32%.

**Figure 6:**
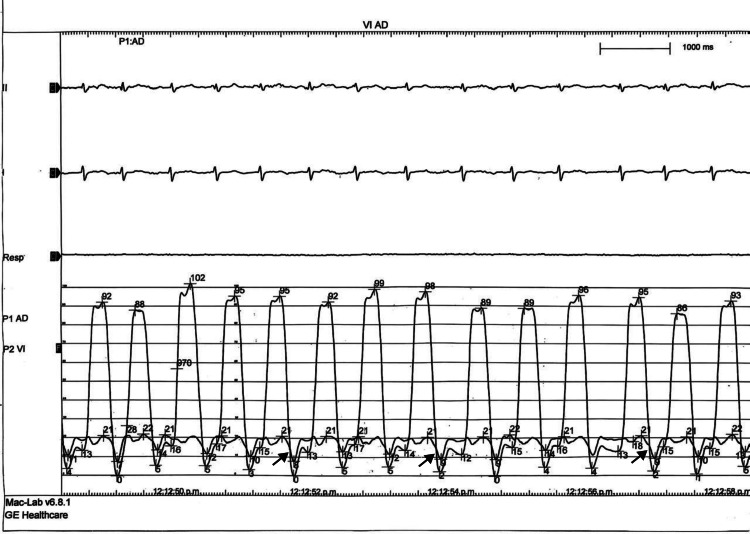
Right and left heart catheterization: left ventricular (LV) and right atrial (RA) pressure tracings Right atrial pressure tracing showing elevated right atrial pressure with a prominent "y" descent (black arrows).

**Figure 7:**
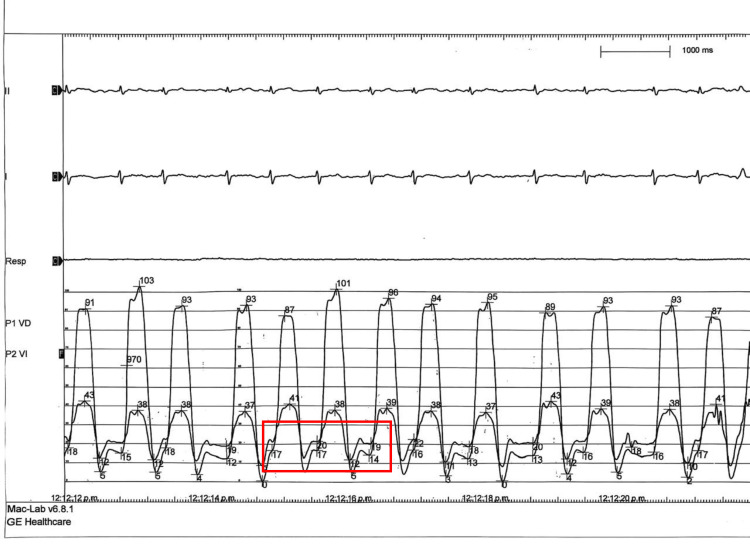
Right and left heart catheterization: left ventricular and right ventricular pressure tracings Dip-and-plateau morphology is observed in both the right ventricle (RV) and left ventricle (LV) pressure tracings (black arrow). Elevation and "equalization" of end-diastolic pressures in both ventricles (red box).

Based on the comprehensive diagnostic evaluation, rare myopericardial involvement was confirmed in the context of suspected extrapulmonary pleural TB. Given additional clinical and imaging findings suggestive of constrictive physiology, right and left heart catheterization was performed (Figures 6, 7). Hemodynamic assessment demonstrated features consistent with constrictive pericarditis, including a dip-and-plateau (square root) pattern in both right and LV pressure tracings and a right ventricular end-diastolic pressure (RVEDP) exceeding one-third of the pulmonary artery systolic pressure (PASP). Hemodynamic measurements revealed an LV systolic pressure of 91 mmHg, a diastolic pressure of 5 mmHg, and a left ventricular end-diastolic pressure (LVEDP) of 15 mmHg. RV pressures were 43/12 mmHg, with an RVEDP of 20 mmHg, resulting in a 5 mmHg difference between LVEDP and RVEDP. Pulmonary artery pressures were 42/22 mmHg, with a mean pulmonary artery pressure (mPAP) of 28 mmHg. Right atrial pressure was elevated at 13 mmHg. Coronary angiography demonstrated no evidence of obstructive coronary artery disease.

A diagnosis of myopericarditis with constrictive physiology associated with probable pleural TB was established. Therapeutic options, including pericardiectomy, were considered. However, following a multidisciplinary discussion and in view of the high surgical risk related to severe ventricular dysfunction, atrial fibrillation, protein-calorie malnutrition, and active, untreated extrapulmonary TB, an initial conservative medical strategy was pursued.

Antituberculous therapy was initiated using a fixed-dose combination containing isoniazid (75 mg), rifampicin (150 mg), pyrazinamide (400 mg), and ethambutol (300 mg), in combination with guideline-directed medical therapy for heart failure, including angiotensin receptor-neprilysin inhibitors (ARNIs), beta-blockers, sodium-glucose cotransporter 2 (SGLT2) inhibitors, spironolactone, digoxin, and loop diuretics.

The patient was discharged with a plan for close follow-up at the heart failure and cardiomyopathy clinic to reassess the potential need for surgical intervention. At the three-month follow-up, he demonstrated mild functional improvement, corresponding to New York Heart Association (NYHA) functional class II, with a significant reduction in N-terminal pro-B-type natriuretic peptide (NT-proBNP) levels to 4,617 pg/mL, resolution of ascites, and only mild residual lower-extremity edema. Optimized medical therapy was continued in accordance with the patient’s preference. Although an extended 12-month antituberculous regimen was initially considered due to the severity of extrapulmonary involvement, subsequent clinical improvement during follow-up supported continuation of the standard six-month regimen, which was ongoing at the time of manuscript submission.

## Discussion

We present a case of myopericarditis with constrictive physiology associated with probable pleural TB, a form of extrapulmonary TB. *M. tuberculosis* remains a major global health concern. According to the Global TB Report 2025, more than 10 million people continue to develop TB each year, with over 1 million deaths annually, making TB the leading cause of death from a single infectious agent and one of the top 10 causes of death worldwide [3]. In Mexico, TB remains a significant public health concern, with over 28,000 notified cases reported in 2023, corresponding to a national incidence of approximately 19-29 cases per 100,000 population, with marked geographic and socioeconomic heterogeneity [3].

Cardiac involvement in TB is rare, occurring in less than 2% of all TB cases, and most commonly affects the pericardium [4-6]. In a review of cases reported between 1955 and 2020, López et al. found that pericarditis accounted for approximately 2%-5% of all cardiac TB presentations [4]. Despite its rarity overall, tuberculous pericarditis remains a major cause of pericardial disease in TB-endemic regions. Recent reviews report that tuberculous pericarditis accounts for approximately 50%-70% of cases of effusive pericarditis in HIV-negative patients and more than 90% in HIV-positive patients in high-burden settings, whereas it represents only about 4% of cases in non-endemic regions. In contrast, idiopathic, post-surgical, and radiation-related etiologies predominate in developed settings. Tuberculous pericarditis has an overall mortality rate of 17%-40% at six months after diagnosis [7].

This marked epidemiological divergence provides important context for the present case, in which pleural TB with associated myopericardial involvement was strongly suspected. Myocardial involvement by *M. tuberculosis* is uncommon and may result from direct extension of pericardial disease, lymphatic spread from mediastinal lymph nodes, or hematogenous dissemination from a distant infectious focus [4]. These pathways may explain the coexistence of myocarditis with pericardial inflammation and subsequent constrictive physiology in advanced extrapulmonary disease.

Constrictive pericarditis is characterized by progressive exertional dyspnea, fatigue, persistent lower-extremity edema, and abdominal distension with ascites, along with other signs of right-sided heart failure [1-6]. The thick and noncompliant pericardium traps the heart within, impairing diastolic filling and resulting in significant systemic venous congestion. A diagnosis of constrictive pericarditis is established by the presence of signs and symptoms of right-sided heart failure, combined with evidence of impaired diastolic filling due to pericardial constriction, as demonstrated by one or more imaging modalities, including echocardiography, CT, CMR, or cardiac catheterization [8,9].

Myocardial involvement by *M. tuberculosis* often poses a diagnostic challenge, requiring integration of clinical history, laboratory findings, and advanced imaging studies. The clinical diagnosis of myocarditis is supported by elevated cardiac biomarkers such as troponin I or T and creatine kinase-MB, along with reduced left ventricular function documented by echocardiography or CMR [10].

Echocardiography may demonstrate a thickened pericardium, interventricular septal bounce during inspiration, respiratory variation in early mitral inflow velocity (E-wave), annulus paradoxus, peak e’ > 8.0 cm/s on tissue Doppler [9], a dilated inferior vena cava, and large bilateral pleural effusions. However, echocardiography can have limitations, such as a restricted acoustic window or the presence of arrhythmias (e.g., atrial fibrillation), which may reduce the sensitivity of some diagnostic criteria. In such cases, other imaging modalities such as CT or CMR may be required.

CMR is an invaluable diagnostic tool that provides comprehensive tissue characterization in suspected perimyocarditis. According to the updated Lake Louise Criteria, the diagnosis integrates parametric mapping techniques with LGE assessment. It requires at least one T2-based criterion, such as increased signal intensity on T2-weighted sequences or elevated myocardial T2 values, indicating active inflammation or myocardial edema, together with at least one T1-based criterion, such as elevated native T1 values, increased extracellular volume, or LGE in a non-ischemic distribution. This non-ischemic pattern typically involves the subepicardial or mid-myocardial layers, most often affecting the lateral and inferior walls of the left ventricle, in a patchy distribution that does not correspond to coronary artery territories. In perimyocarditis, CMR may also demonstrate pericardial thickening, pericardial enhancement, and pericardial effusion [11]. Pericardial enhancement is considered a marker of active pericardial inflammation.

In the present case, the patient met the Lake Louise Criteria, demonstrating increased native T1 mapping values in the inferolateral segment together with LGE on T2-weighted sequences, showing a subepicardial pattern in the lateral wall of the left ventricle. Pericardial involvement was also present, characterized by pericardial thickening and positive LGE.

Despite advances in multimodality imaging, invasive catheterization may still be required. In our case, cardiac catheterization was necessary to confirm enhanced ventricular interaction and constrictive physiology through analysis of LV and RV hemodynamics. Typical findings include (a) elevation and equalization of end-diastolic pressures in both ventricles (RVEDP and LVEDP), typically with a difference ≤5 mmHg; (b) the classic "square root" or "dip-and-plateau" pattern in ventricular pressure curves during diastole, reflecting rapid early filling followed by an abrupt plateau due to pericardial constraint; (c) pronounced ventricular interdependence, with inspiratory increases in RV diastolic pressure accompanied by decreases in LV diastolic pressure, and the reverse during expiration; and (d) elevated right atrial pressure, often with a prominent "y" descent on the pressure tracing [8,12]. The relationship between RVEDP and PASP has also been described; an RVEDP greater than one-third of PASP (RVEDP/PASP > 1/3) supports a diagnosis of constrictive pericarditis, although this parameter lacks absolute sensitivity and specificity and should be interpreted in the context of other hemodynamic and clinical findings [13].

Our patient fulfilled these hemodynamic criteria for constrictive physiology, demonstrating a prominent "y" descent, a dip-and-plateau pattern, equalization of biventricular end-diastolic pressures with an average difference of 5 mmHg, and an RVEDP (20 mmHg) greater than one-third of PASP (42 mmHg). Combined with multimodality imaging findings, these results confirmed the diagnosis of myopericarditis with constrictive physiology.

Together, multimodality imaging allowed sequential anatomical, functional, and hemodynamic characterization, which was essential for confirming the diagnosis in this complex perimyocardial presentation.

The gold standard for the definitive diagnosis of pleural TB is the detection of *M. tuberculosis* in pleural fluid or pleural tissue, or the demonstration of caseating granulomas on pleural biopsy, ideally with the presence of acid-fast bacilli (AFB) [14]. However, achieving this diagnostic gold standard remains a persistent challenge in pleural TB due to its paucibacillary nature. A similar diagnostic limitation applies to tuberculous pericarditis, in which microbiological confirmation from pericardial fluid or tissue is frequently difficult to obtain, and diagnosis often relies on a combination of clinical presentation, epidemiological context, biochemical markers, imaging findings, and therapeutic response.

TB pleural effusions are typically exudates. Fluid LDH and protein are elevated in over 75% of cases, and fluid glucose may be low compared with serum [14]. Nucleic acid amplification tests (NAATs) can rapidly detect Mtb-specific nucleic acid sequences in clinical specimens. Although Xpert MTB/RIF is highly specific for the detection of* M. tuberculosis *in pleural fluid, its diagnostic sensitivity in pleural TB is limited [15]. A large meta-analysis including 4,207 patients from both high- and low-prevalence settings reported a pooled sensitivity of approximately 51% when compared with pleural fluid culture, decreasing to 18% when a composite reference standard was used, while specificity remained consistently high (>98%) [15]. Pleural fluid ADA levels above 40 U/L are widely used as a diagnostic threshold for pleural TB and appear to be largely unaffected by HIV status [14]. A large meta-analysis including over 27,000 patients reported high pooled sensitivity (≈92%) and specificity (≈90%) for ADA in the diagnosis of pleural TB, although substantial heterogeneity and risk of bias across studies limit universal applicability [16]. Interpretation of ADA levels should therefore consider pre-test probability, as the positive predictive value is high in TB-endemic regions but declines in low-prevalence settings. Importantly, elevated ADA levels are not specific to TB, and very high values (>250 U/L) should prompt consideration of alternative diagnoses such as empyema or lymphoma [14].

Although microbiological confirmation of *M. tuberculosis* was not achieved in this case, the overall diagnostic evidence strongly supported TB as the underlying etiology. The presence of an exudative pleural effusion with markedly elevated ADA levels, exclusion of malignancy and alternative infectious causes, epidemiological risk in a TB-endemic region, and a clinical presentation consistent with advanced extrapulmonary disease collectively supported a diagnosis of probable pleural TB. Importantly, the coexistence of myopericarditis with constrictive physiology further strengthened this diagnostic consideration, as TB remains the most common cause of constrictive pericarditis worldwide, particularly in endemic settings. Taken together, these findings provided sufficient justification for antituberculous therapy despite the absence of direct microbiological isolation.

Medical therapy for tuberculous pericarditis can be effective in preventing progression to constrictive pericarditis. Antituberculosis antibiotics can reduce the risk of constriction from >80% to <10% [1,7]. Corticosteroids are an important adjunctive therapy in tuberculous pericarditis and have been shown to significantly reduce the risk of constriction compared to placebo (4.4% vs. 7.8%; hazard ratio, 0.56; 95% CI, 0.36-0.87; p = 0.009) [2]. However, our patient already presented with established constrictive physiology. Current evidence indicates that in tuberculous pericarditis with established constriction, standard antituberculosis therapy remains essential to control the infection and prevent further disease progression [17]. The role of corticosteroids in patients with established constriction remains controversial, and there is no robust evidence to support their routine use in this setting.

According to the ESC Guidelines for the Diagnosis and Management of Pericardial Diseases, pericardiectomy is the standard treatment for patients with chronic constrictive pericarditis who have persistent and severe symptoms, such as those in NYHA class III or IV. However, surgery should be considered cautiously in patients with mild or very advanced disease and in those with myocardial or significant renal dysfunction [7]. Patients with end-stage constrictive pericarditis derive little or no benefit from pericardiectomy, and operative risk is markedly elevated. End-stage manifestations include cachexia, atrial fibrillation, low cardiac output (cardiac index <1.2 L/min/m²) at rest, hypoalbuminemia, and/or impaired hepatic function. Pericardiectomy carries considerable morbidity and mortality; a meta-analysis of 27 studies including 2,114 patients reported an operative mortality of 6.9% and a five-year mortality of 32.7% [18].

This case has several limitations that should be acknowledged. Histopathological confirmation was not obtained, as pleural, pericardial, or myocardial biopsy was not performed, and pericardial fluid analysis was not available. Consequently, direct microbiological or histological evidence of *M. tuberculosis* involving the pericardium or myocardium could not be demonstrated. However, invasive diagnostic procedures were deferred due to the patient’s clinical condition, high procedural risk, and the presence of advanced constrictive physiology. In this context, the diagnosis relied on a comprehensive integration of epidemiological exposure, clinical presentation, biochemical findings, multimodality imaging, exclusion of alternative etiologies, and therapeutic response.

The educational value of this case lies in the rare coexistence of myocarditis and constrictive pericarditis within a clinical context strongly suggestive of extrapulmonary TB with pleural involvement. This presentation highlights the need to maintain diagnostic suspicion in endemic settings and demonstrates how integrating multimodality imaging with invasive hemodynamic assessment can clarify complex perimyocardial disease and guide management in high-risk surgical candidates. Furthermore, it underscores the importance of individualized clinical decision-making in complex presentations of tuberculous perimyocardial disease. While current guidelines recognize pericardiectomy as the definitive treatment for chronic constrictive pericarditis, clinical scenarios involving concomitant myocardial involvement and advanced constrictive physiology are not explicitly addressed. In this particular context, characterized by extensive myopericardial involvement, severe ventricular dysfunction (particularly RV impairment), and high surgical risk, a conservative approach with optimized medical therapy and antituberculous treatment was initially favored, with pericardiectomy deferred rather than excluded, based on clinical response and multidisciplinary assessment. Long-term follow-up remains essential to monitor clinical stability and to reassess candidacy for surgical intervention as the patient's condition evolves.

## Conclusions

Tuberculous myopericarditis with constrictive physiology is a rare but potentially severe manifestation of extrapulmonary TB. Early recognition through comprehensive evaluation, including multimodality imaging and invasive hemodynamic assessment, is crucial for accurate diagnosis. In selected high-risk surgical patients, optimal medical therapy, guided by a multidisciplinary heart team and aligned with patient preferences, can provide early clinical and functional improvement while minimizing the risks associated with pericardiectomy. Short-term follow-up of our patient demonstrated symptomatic improvement and reduction of systemic congestion, supporting an individualized, conservative management strategy. This case reinforces the need for tailored therapeutic approaches and close longitudinal follow-up in complex presentations of tuberculous pericardial disease.

## References

[1] Mayosi BM, Burgess LJ, Doubell AF (2005). Tuberculous pericarditis. Circulation.

[2] Welch TD, Oh JK (2017). Constrictive pericarditis. Cardiol Clin.

[3] (2026). Global tuberculosis report 2025. https://www.who.int/publications/i/item/9789240116924.

[4] López-López JP, Posada-Martínez EL, Saldarriaga C (2021). Tuberculosis and the heart. J Am Heart Assoc.

[5] Marcu DT, Adam CA, Mitu F (2023). Cardiovascular involvement in tuberculosis: from pathophysiology to diagnosis and complications—a narrative review. Diagnostics (Basel).

[6] Fairley CK, Ryan M, Wall PG, Weinberg J (1996). The organism reported to cause infective myocarditis and pericarditis in England and Wales. J Infect.

[7] Schulz-Menger J, Collini V, Gröschel J (2025). 2025 ESC guidelines for the management of myocarditis and pericarditis. Eur Heart J.

[8] Talreja DR, Nishimura RA, Oh JK, Holmes DR (2008). Constrictive pericarditis in the modern era: novel criteria for diagnosis in the cardiac catheterization laboratory. J Am Coll Cardiol.

[9] Welch TD, Ling LH, Espinosa RE (2014). Echocardiographic diagnosis of constrictive pericarditis: Mayo Clinic criteria. Circ Cardiovasc Imaging.

[10] Michira BN, Alkizim FO, Matheka DM (2015). Patterns and clinical manifestations of tuberculous myocarditis: a systematic review of cases. Pan Afr Med J.

[11] Drazner MH, Bozkurt B, Cooper LT (2025). 2024 ACC expert consensus decision pathway on strategies and criteria for the diagnosis and management of myocarditis: a report of the American College of Cardiology solution set oversight committee. J Am Coll Cardiol.

[12] Geske JB, Anavekar NS, Nishimura RA, Oh JK, Gersh BJ (2016). Differentiation of constriction and restriction: complex cardiovascular hemodynamics. J Am Coll Cardiol.

[13] Sorajja P, Borlaug BA, Dimas VV (2017). SCAI/HFSA clinical expert consensus document on the use of invasive hemodynamics for the diagnosis and management of cardiovascular disease. Catheter Cardiovasc Interv.

[14] McNally E, Ross C, Gleeson LE (2023). The tuberculous pleural effusion. Breathe (Sheff).

[15] Kohli M, Schiller I, Dendukuri N (2018). Xpert(®) MTB/RIF assay for extrapulmonary tuberculosis and rifampicin resistance. Cochrane Database Syst Rev.

[16] Aggarwal AN, Agarwal R, Sehgal IS, Dhooria S (2019). Adenosine deaminase for diagnosis of tuberculous pleural effusion: a systematic review and meta-analysis. PLOS One.

[17] Saukkonen JJ, Duarte R, Munsiff SS (2025). Updates on the treatment of drug-susceptible and drug-resistant tuberculosis: an official ATS/CDC/ERS/IDSA clinical practice guideline. Am J Respir Crit Care Med.

[18] Tzani A, Doulamis IP, Tzoumas A (2021). Meta-analysis of population characteristics and outcomes of patients undergoing pericardiectomy for constrictive pericarditis. Am J Cardiol.

